# Discontinuation of biologic DMARDs in non-systemic JIA patients: a scoping review of relapse rates and associated factors

**DOI:** 10.1186/s12969-022-00769-5

**Published:** 2022-12-05

**Authors:** Job Gieling, Bart van den Bemt, Esther Hoppenreijs, Ellen Schatorjé

**Affiliations:** 1 Department of Pediatric Rheumatology, Pediatrics, Radboud University Medical Center, Nijmegen, the Netherlands; 2 Departments of Pharmacy, Sint Maartenskliniek / Radboud University Medical Center, Nijmegen, the Netherlands; 3 Department of Pediatric Rheumatology, Pediatrics, Sint Maartenskliniek / Radboud University Medical Center, Nijmegen, the Netherlands

**Keywords:** Juvenile idiopathic arthritis, Biologic therapy, Biologic disease-modifying antirheumatic drugs, Tapering, Relapse, Flare

## Abstract

**Background:**

Biologic disease-modifying antirheumatic drugs (bDMARDs) have changed the treatment of juvenile idiopathic arthritis (JIA) patients notably, as bDMARDs enable substantially more patients to achieve remission. When sustained remission is achieved, tapering or even discontinuation of the bDMARD is advocated, to reduce side effects and costs. However, when and how to discontinue bDMARD therapy and what happens afterwards, is less known.

**Objectives:**

With this scoping review we aim to collect available data in current literature on relapse rate, time to relapse (TTR) and possible flare associated variables (such as time spent in remission and method of discontinuation) after discontinuing bDMARDs in non-systemic JIA patients.

**Methods:**

We performed a literature search until July 2022 using the Pubmed database. All original studies reporting on bDMARD discontinuation in non-systemic JIA patients were eligible. Data on patient- and study characteristics, the applied discontinuation strategy, relapse rates and time to relapse were extracted in a standardized template.

**Results:**

Of the 680 records screened, 28 articles were included in this review with 456 non-systemic JIA patients who tapered and/or stopped bDMARD therapy. Relapse rate after discontinuation of bDMARDs, either abruptly or following tapering, were 40–48%, 36.8–45.0% and 60–78% at 6, 8 and 12 months respectively. Total relapse rate ranged from 26.3% to 100%, with mean time to relapse (TTR) of 2 to 8.4 months, median TTR 3 to 10 months. All studies stated a good response after restart of therapy after flare.

JIA subtype, type of bDMARD, concomitant methotrexate use, treatment duration, tapering method, age, sex, and time in remission could not conclusively be related to relapse rate or TTR. However, some studies reported a positive correlation between flare and antinuclear antibodies positivity, younger age at disease onset, male sex, disease duration and delayed remission, which were not confirmed in other studies.

**Conclusion:**

Flares seem to be common after bDMARD discontinuation, but little is known about which factors influence these flares in JIA patients. Follow up after discontinuation with careful registration of patient variables, information about tapering methods and flare rates are required to better guide tapering and/or stopping of bDMARDs in JIA patients in the future.

**Supplementary Information:**

The online version contains supplementary material available at 10.1186/s12969-022-00769-5.

## Introduction

Juvenile idiopathic arthritis (JIA) is the most common rheumatic disease in childhood and an important cause of short- and long-term disability [1]. Biologic disease-modifying antirheumatic drugs (bDMARDs) are proven effective in JIA patients and have been successfully implemented in the standard treatment regime [2–5]. Still bDMARDs are costly and come with (dose-dependent) side effects [2, 6–8]. Reducing or stopping bDMARDs (when disease activity is low) might therefore be a valuable strategy to reduce side effects (in particular infections) and costs. Dose reduction in adult population of rheumatoid arthritis (RA) patients has been proven to be successful, yet discontinuation is not recommended, as many adult RA patient flare after discontinuation [9–13]. However, some JIA patients may recover spontaneously, with studies reporting over 50% of patients being in clinical remission off medication, 30 years after disease onset. Children therefore have more favorable outcomes than RA patients, this suggests that discontinuation might be a viable option in JIA patients [14, 15]. However, the evidence about the success rate of discontinuation bDMARDs in JIA is not summarized yet. Furthermore when, how and in whom to discontinue bDMARDs in JIA is not known.

The aim of this review was to conduct a scoping review of all available evidence on relapse rates and relapse associated variables after bDMARD discontinuation in children with non-systemic JIA.

## Methods

This review was guided by the Preferred Reporting Items for Systematic reviews and Meta-Analyses extension for Scoping Reviews Checklist, checklist and additional information can be found in supplement 1 [16].

To better guide bDMARD discontinuation in children with non-systemic JIA, the following key questions were formulated;

1. What is the relapse rate and time to relapse after discontinuation of a bDMARD?
2. Which factors are associated with flares after treatment discontinuation;
  - Are there differences in flare rate between age, type of bDMARD or JIA subgroups?
  - Does disease severity (e.g., time in remission, treatment or disease duration, time to remission) affect flare rate or flare severity after bDMARD discontinuation?
  - What is the difference between flare rate/flare severity when patients are tapered before bDMARD discontinuation compared to abrupt discontinuation?
  - Does the use of multiple bDMARDs or concomitant therapy affect flare rate or flare severity, and when concomitant therapy is used, is there a preference in stopping one before the other?

### Search strategy and eligibility of the studies

Eligible articles were identified by a systematic search of PubMed/MEDLINE database. The initial search was performed in April 2021, and was repeated in July 2022 to include articles published between April 2021 and July 2022. Different MeSH terms and synonyms for JIA, bDMARD, Abatacept, Adalimumab, Etanercept, Golimumab, Infliximab, and Tocilizumab were used. The complete list of used search terms and complete search strategy are set out in supplement 2.

Titles and abstracts were independently screened by two researchers. Any discrepancies were resolved by consensus agreement between the two investigators. After reading title and abstract, original articles written in English, Dutch, French or German reporting on bDMARD discontinuation in non-systemic JIA patients were selected. Review papers, guidelines, case reports and studies focusing on systemic onset JIA (soJIA), uveitis and psoriasis were excluded. Subsequently, after reading the full text, studies lacking data on previously mentioned key questions, studies with overlapping data and studies of which no full text article was available were excluded. Using references mentioned in relevant articles, a further manual search for additional articles was conducted.

### Data selection

Data charting was primarily performed by one researcher. When data was ambiguous, interpretation of this data was verified by a second researcher. Data regarding study design, number of patients undergoing bDMARD discontinuation, mode of discontinuation, medication use, disease course, flare rate and response to therapy after flare were extracted from each eligible study. We defined patients as having flared when the study described the patient as having flared, relapsed, when therapy was restarted or when the patient was no longer defined as being in remission by the study. Additionally, patient demographics of the relevant study groups were noted, if provided. Statements about possible correlations to flare were obtained from text or extracted from presented tables or supplements when possible. After the outcome measures of the eligible studies were tabulated, they were analyzed with a purely descriptive approach.

## Results

### Search results

**Fig. 1:**
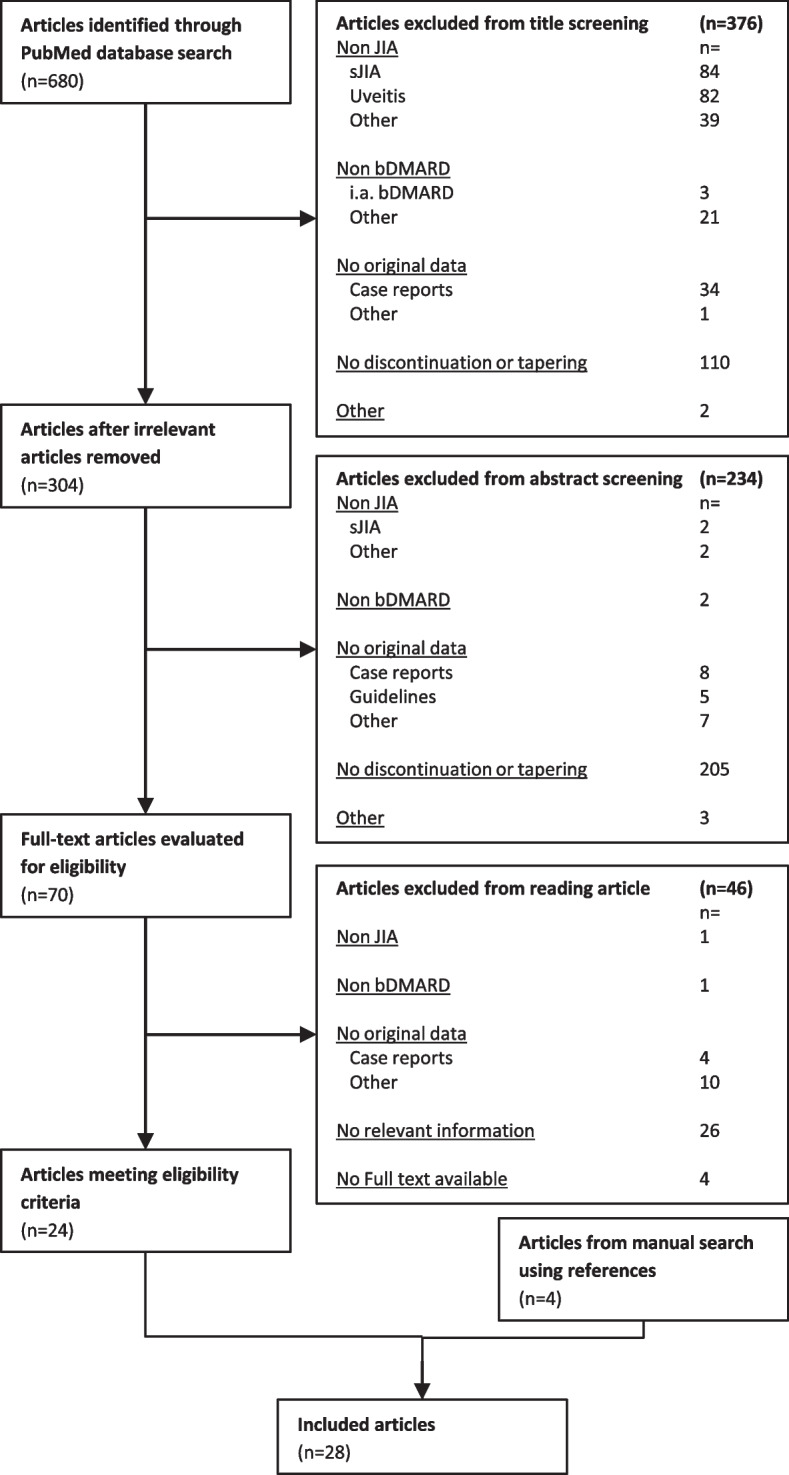
Article selection

**Fig. 2:**
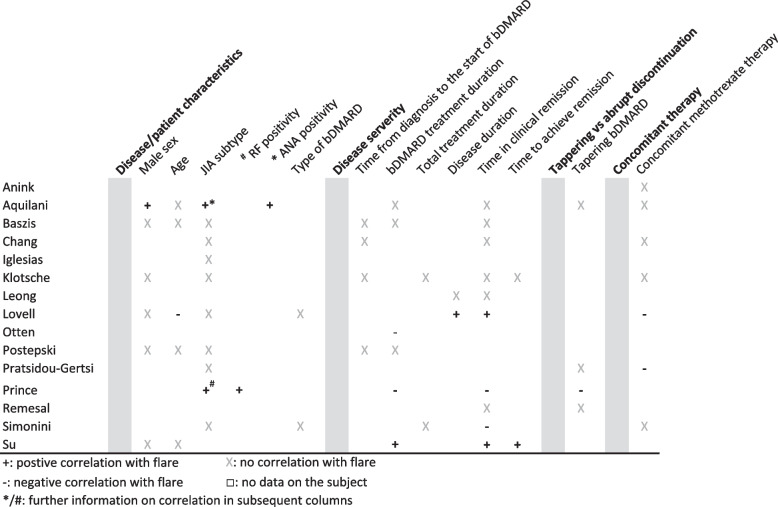
Flare associated variables

Literature screening identified a total of 680 articles. Of these 28 articles, published in 12 different journals from 2008 to 2021, were included after full text analysis, as depicted in Fig. 1. The number of prospective and retrospective studies were equally split. Three studies used either a specific selection of patients from the BiKeR registry [17] or a combination of registries including the BikeR registry, so some overlap in data between these studies could not be excluded [18–20]. Etanercept (ETN) was the most frequently studied bDMARD. Flare rate after bDMARD discontinuation was a primary outcome measure in 12 articles. Of the remaining articles, flare rate or flare associated variables were mentioned in text or figures, but were not the primary outcome. Fourteen articles (456 patients) reported on non-systemic JIA patients discontinuing bDMARDs, whereas 14 articles (730 patients) included both systemic and non-systemic JIA patients, without adequate discrimination between the two subgroups by the authors. Therefore, these outcome variables may also contain results of patients with soJIA. However, their absolute contribution to the study group was deemed to be low (Table 2). Relapse rates are displayed for both groups separately (non-systemic JIA (Table 1) and mixed systemic/non-systemic JIA (Table 2). The percentage of soJIA patients are also shown in the table. Figure 2 shows the correlations of variables with flares as stated in the different articles.

**Table 1:** Relapse rate in non-systemic JIA patients after withdrawal of bDMARD

Main author	Anink	Giménez- Roca	Lovell	Leong	Aquilani	Chang			Hissink Muller	Prince	Foeldvari	Iglesias	Windschall	Southwood	Otten
[Ref]	[18]	[21]	[22]	[23]	[24]	[25]			[26]	[27]	[28]	[29]	[19]	[30]	[31]
n=	26	6	106	20	110	25^e^	17^f^	7^g^	19	14	22	18	24	5	6
Medication type	Etanercept	Etanercept	Etanercept	Etanercept	Etanercept	Etanercept			Etanercept	Etanercept	Etanercept	Etanercept	Etanercept	Etanercept	Etanercept
		Adalimumab	Adalimumab	Adalimumab		Adalimumab						Adalimumab			
			Infliximab	Infliximab		Infliximab						Infliximab			
Time in inactive disease, months	at least 6	12	at least 6	at least 6		unknown					unknown		at least 18^j^	unknown	unknown^k^
*mean (range)*									5.8 (3-6)	8 (0-35)		12.2^h^			
*median (range)*					14.6^c^										
Follow up duration, months	6	7	8	8	12	at least 6			unknown	unknown	unknown	unknown	unknown	unknown	unknown
% relapse after withdrawal	46.2%	100%	36.8%	45%	60%	78%^e^	76%^f^	78%^g^	26.3%	57.1%	59.1%	77.80%	50%	60%	83.3%
Time to relapse, months															
*mean (range)*			7^a^									3.0^i^	8.4 (4-72)		2 (0,7-10)
*median (range)*			8.3^b^		4.3^d^				3 (3-7.5)	2,6 (0,4-27)	7			10 (5-34)	
% relapse after 6 months	46.2%				40%	47%^e^	48%^f^	31%^g^							
% relapse after 7 months		100%													
% relapse after 8 months			36.8%	45%											
% relapse after 12 months					60%	78%^e^	76%^f^	78%^g^							

^a^SEM 0.32

^b^median CI (7.8-8.6)

^c^IQR (11.1-21.3)

^d^IQR 2.5 -6.4

^e^TNFi +MTX; TNFi withdrawl first

^f^TNFi + MTX; TNFi MTX withdrawl first

^g^TNFi monotherapy

^h^SD 7.2 hSD 2.0

^i^SD 11,3

^j^In 23 of the 24 patients time of inactive disease was longer than 18 months

^k^Treatment duration 22 months (13-55)

**Table 2:** Relapse rate in mixed systemic/non-systemic JIA patients after withdrawal of bDMARD

Main author	Baszis	Remesal	Postępski	Simonini	Romano	Vidqvist	Pratsidou-Gertsi & Trachana	Anink	Otten	Su	Tynjälä	Klotsche
[ref]	[32]	[33]	[34]	[35]	[36]	[37]	[38, 39]	[40]	[41]	[42]	[43]	[20]
n=	136	26	39	135	27	19	13	18	39	30	27	209
% of soJIA patients in withdrawl-group	unknown	8	18	9	unknown	unknown	27^e^	unknown	unknown	10	unknown	unknown
% of soJIA patients in entire study^a^	12	NA	NA	NA	17	1	NA	28	18	NA	6	6
Medication type	Etanercept	Etanercept	Etanercept	Etanercept	Etanercept	Etanercept	Etanercept	Etanercept	Etanercept	Etanercept	Etanercept	Etanercept
	Adalimumab			Adalimumab	Adalimumab	Adalimumab					Infliximab	
	Infliximab			Infliximab	Infliximab	Infliximab						
				^d^								
Time in inactive disease, months		at least 6^c^		at least 6	at least 6	unknown	at least 6	unknown	unknown	at least 6	unknown	unknown
*mean (range)*			21.3 (4-42)									
*median (range)*	6.1 (0-67.9)											
Follow up duration, months	^b^				unknown	unknown	at least 12				unknown	
*mean (range)*		21 (5-44.5)	25.4 (16-60)	6 (3-109)				(10-78)	13.4^f^	26		46.8^g^
% relapse after withdrawal	68%	69.2%	69.2%	75.6%	48.1%	unknown	84.6%	77.8%	38.5%	56.7%	55.6%	77%
Time to relapse, months												
*mean (range)*	5.8 (0.6-15.9)		14.2 (1-60)			18 (0-108)						
*median (range)*					6.6 (2.4-28.6)		3 (1-15)	9 (1-69)				
% relapse after 3 months	25%											
% relapse after 6 months	50%	50%	38.5%									
% relapse after 12 months	68%	61%		68.9%								

*NA* not applicable

^a^In case the portion of sJIA patients in the withdrawl study was not known

^b^follow up was > 12 months in 71% of patients

^c^24 out of 26 were in clinical remission on medication

^d^as well as anakinra (in 7 patients), rituximab and abatacept (both in 1 patient)

^e^In 2 patients JIA subtype is unknown [43]

^f^IQ 5.3 - 24.7

^g^SD 3.5 years

### How many flares are seen after discontinuation of a bDMARD?

**Fig. 3:**
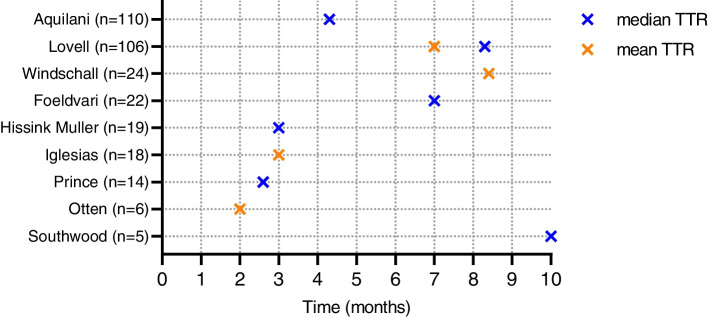
Time to relapse (TTR) in non systemic JIA patients after withdrawl of bDMARDs

Table 1 and the figure in supplement 3 display the relapse rate of the non-systemic group. Relapse rate at 6 and 12 months were 40–48%, and 60–78% respectively [18, 24, 25]. One study reported a relapse rate of 100% at 7 months based on a small study population of 6 patients [21]. Total relapse rate, at different follow-up durations, ranged from 26.3% to 100%, with mean time to relapse (TTR) of 2 to 8.4 months and median TTR of 3 to 10 months [18, 19, 21–31] as shown in Fig. 3.

As shown in Table 2 relapse rates in the group in which soJIA could not be excluded were comparable to rates in the non-systemic JIA group, with relapse rates at 6, 12 and 48 months of 38.5–50%, 61–63.9% and 77% [20, 32–35]. Total relapse rate ranged from 38.5% to 84.6% with a mean TTR of 5.8 to 18 months, and median TTR of 3 to 9 months [32–42].

All studies reported a good response to restarting therapy after a flare, with most studies mentioning a prompt return to inactive disease. However the definition of a good response and the therapy started after flare were not always well defined [20, 26, 27, 29, 31, 33, 34, 38, 43].

### Are there differences between age, bDMARD or JIA subgroups?

#### General patient characteristics

The impact of sex and age on successful discontinuation was contradictory. Where Aquilani reported more flares in men than women (*n* = 110, *p* = 0.02), this was not confirmed by Lovell or Klotsche [20, 22, 24]. With respect to age, the results again were inconclusive. Lovell found an association between younger age and an increased chance of flares, whereas Aquilani did not find age as a discriminatory factor [22, 24]. Other studies stated no significant correlation between age or sex, and flares [32, 34, 42].

#### JIA subtype

Most studies found no correlation between JIA subtype and relapse rate or TTR [20, 22, 25, 29, 32, 34, 35, 38], however two studies described higher relapse rate in ANA or Rheumatoid factor (RF) positive patients. In one of them, a significant difference in flare rate was seen between the ANA positive and negative JIA groups, noting 48 out of 71 patients flared versus 18 out of 39 patients respectively (*p* = 0.047). Flares were never uveitis-only [24]. The other study stated RF to be negatively related with sustained remission, however this study included no more than 2 RF positive patients [27].

#### bDMARD

Only two studies reported on the relation between different bDMARDs and flares concluding that the type of biologic treatment was not a predictor of long-lasting remission [22, 35].

### Does disease severity (e.g., treatment or disease duration, time in remission, time to achieve remission) affect flare rate or flare severity?

#### Time from diagnosis to start of bDMARD treatment, treatment duration and disease duration

Time from diagnosis to the start of bDMARD treatment [20, 25, 32, 34] and total treatment duration [20, 35] were not related to relapse rate or TTR. Some studies reported contradictory results regarding bDMARD treatment duration such as Prince and Otten reporting on a (trend towards) higher flare rate after a shorter duration of ETN treatment (2.1 vs. 3.5 years *p* = 0.21 and 2.4 vs. 3.8 years *p* = 0.03 resp.) whereas Su showing the opposite (15.8 vs 6.1 months *p* = 0.0006) [27, 41, 42]. A few other studies found no difference in flare rate at all, when comparing duration of bDMARD therapy [20, 24, 32, 34]. Shorter total disease duration was linked to fewer flares in Lovell (*n* = 105, Hazard Ratio (HR) 1.12 *p* < 0.01), but not in Leong (*n* = 39) [22, 23].

#### Time in clinical remission

Contradictory data was also found regarding the relationship between time in clinical remission before discontinuation and relapse rate/TTR. Most articles report no association between time in clinical remission and flare [20, 23–25, 32, 33]. Lovel found longer time in remission to be a predictor of more frequent flares with a HR 1.16 (*p* = 0.04), as did Su who observed a longer period of clinical inactive disease in their relapse group (8.4 vs. 4.2 months, *p* = 0.046) [22, 42]. Yet Simonini found a clinical remission of longer than 2 years to be linked to a reduction in flares (*p* < 0.002) [35]. The same link between long clinical remission and number of flares was seen by Prince, however their data was heavily skewed by a disproportionately long clinical remission in there soJIA subgroup. Therefore, conclusions regarding non-soJIA patients could not reliably be made on the basis of this specific study [27].

#### Time to achieve remission

Su noted delayed remission to be a risk factor for flare [42]. The duration from starting ETN to achieving remission was 8 months in the non-relapsing group vs. 14.9 months in the relapsing group, resulting in a HR of 1.12 (*p* = 0.0004) when remission is delayed by one month. This is in contrast to Klotsche who found response to ETN in the first 6 months not to be related to reoccurrence of active disease after ETN discontinuation [20].

### Does tapering before discontinuation affect flare rate or flare severity, when compared to abrupt discontinuation?

Prince found 4 out of 5 non-systemic JIA patients flared after abrupt discontinuation versus 4 out of 9 in whom ETN was tapered before stopping [27]. Other studies reported no differences in relapse rate and TTR between tapered and abrupt discontinuation groups [24, 33, 38]. No data was found on the correlation between the tapering method (e.g., dose reduction or interval prolongation) and relapses.

Two studies did not identify loss of effectiveness after halving the standard dose of ETN from 0.4 mg/kg to 0.2 mg/kg, showing a low relapse rate after tapering. However, bDMARDs were not completely stopped in these studies [44, 45].

### Does the use of multiple bDMARDs or concomitant therapy affect flare rate or flare severity, and when concomitant therapy is used is there a preference in stopping one before the other?

No data was found on the use of multiple bDMARDs in relation to flares.

Concomitant methotrexate (MTX) therapy did not affect the number of flares after discontinuation of TNF inhibitor (TNFi) therapy in most studies [18, 22, 24, 35]. Nonetheless, Lovell reported a decreased risk of flare in concomitant MTX users when compared to non MTX users with a HR of 11.6 (95%CI 1.20, 112.78), but this was seen only in a subgroup who discontinued adalimumab (ADA), not in other bDMARD users [22]. Similarly, Pratsidou-Gertsi found a shorter TTR when MTX was continued after ETN discontinuation (8 vs 2.5 months, *p* = 0.04, *n* = 11) [38].

Chang compared the relapse rate of JIA patients on TNFi-MTX combination therapy after discontinuation of TNFi or MTX while continuing the other, and found a higher relapse rate after TNFi discontinuation when compared to MTX discontinuation (47%, 78% vs 16% and 19% at 6 and 12 months, *P* < 0.0005). When subsequently TNFi was discontinued in the latter group, relapse rates were consistent with relapse rates of the early discontinuation of TNFi (48% and 76% at 6 and 12 months). Therefore, relapse rate after TNFi discontinuation seemed to be unaffected by MTX use [25].

## Discussion

This review summarizes the current knowledge on relapse rate, time to relapse and possible flare associated variables after discontinuing bDMARDs in non-systemic JIA patients. Sixty to 78% of patients flared within one year after discontinuation, mean time to relapse was 2 to 8.4 months. None of the possible flare associated variables could definitively be linked to flares. Studies reporting a correlation were opposed with multiple studies finding no correlation, or correlations contradicting each other altogether. Comparison of data was further complicated by studies not displaying their data on possible flare associated variables in a verifiable and uniform manner.

A former review by Halyabar et al. also looked at treatment withdrawal in JIA but did not focus specifically on the non-systemic JIA group [46]. As natural flare rates in soJIA are known to be significantly lower compared to other JIA subgroups [47], it is important to look at these groups separately in relation to relapse rates following discontinuation of bDMARDs.

In the adult RA population relapse rates have also been studied. In this population, like in JIA, the treatment goal is to achieve remission soon after onset of RA, followed by the most optimal treatment that results in the lowest possible disease activity, the least adverse events and the lowest costs. This is achieved by tapering and when possible discontinuation of medication. In this adult population tapering of TNFi to a more optimal dose has been shown to be safe and feasible when low disease activity or remission is reached [48, 49]. Furthermore, disease activity guided dose tapering seemed to be non-inferior to continuation of full dose TNFi [50, 51]. This is in line with the studies of Cai and Mori reporting no loss of effectiveness after tapering ETN in a JIA population [44, 45]. Still tapering can be accompanied by (temporary) flaring. Fautrel et al. found a relapse rate of 77% after 18 months in RA patients tapering ADA and/or ETN every 3 months followed by discontinuation, compared to 47% in RA patient continuing ADA and/or ETN in a standard dose [52]. Unfortunately, similar to our findings in the JIA population, no markers for successful tapering have been found [53] and even a multi-biomarker score could not predict successful tapering in adults [54]. Nevertheless, protocolised tapering of TNFi seemed to be cost effective in the RA population [55]. Studies have shown that discontinuation without prior tapering is inferior to full dose continuation in the adult RA population [48]. It is therefore recommended to taper medication when low disease activity is reached in RA patients, thereby identifying a more optimal dose, as well as identifying patients who are able to discontinue their TNFi [11]. It is not yet clear if this recommendation is applicable to the JIA population. One could argue that a more favourable disease course of JIA in general justifies a more liberal tapering policy.

When interpreting the data in this review there are some considerations to keep in mind. First, flares occur frequently in JIA patients in inactive disease, even when medication is not stopped. Guzman collected data of 1146 JIA patients in inactive disease (receiving different forms of therapy) and found 42.5% of patients developing a flare within one year of achieving inactive disease, with 26.6% of patients developing a significant flare, requiring treatment intensification [47]. Therefore, some of the flares reported in the included studies in this review could also be due to the natural course of the disease and not directly related to stopping of medication.

Another aspect that one should consider, is the likely occurrence of selection bias: JIA patients receiving bDMARDs are probably of a more severe subclass and more flares can be expected in this subclass, either in general or after stoppage. This is again illustrated by Guzman, who showed that bDMARD use was associated with an increased risk of flare (HR 1.65).

This was especially true in the earlier years of bDMARD use, when these drugs were preserved for the most severe patients failing all other therapies. Since treatment strategies changed, bDMARDs are given earlier in the disease course and to less severely affected patients, which will likely result in fewer flares. Earlier studies in this review may therefore report higher flare rates than one would find today.

In addition to publication date, different discontinuation and treatment policies between studies should be kept in mind when interpreting data. For example, *time in clinical remission* could be longer because of cautious discontinuation policies in general, or as a result of a decision to withhold discontinuation in a specific patient due to severity of the disease in this patient. These different reasons for a longer *time in clinical remission* could very well explain the contradictory statements of correlation of *time in clinical remission* and flare made by Prince, Simonini, Su and Lovell [22, 27, 35, 42]. Likewise, selection bias could be an explanation for other correlations found in other flare associated variables. Health professionals could have been more inclined to prescribe concomitant MTX to more severe JIA patients and consequently, more flares could have been found by Lovell or Pratsidou-Gertsi [22, 38]. Unfortunately, the articles did not present enough information to verify these potential explanations.

The most important limitation of this review is that more than half of the selected studies did not select flare rate as their primary outcome. Therefore data was not always sufficiently powered and statements on flare associated variables could not always be verified numerically. Furthermore, data regarding patients lost to follow up was missing in these studies. Additionally, even though we excluded soJIA in order to make the study group more homogeneous, JIA still is a heterogenous disease and general statements are therefore difficult to make. Finally, the follow up in the studies rarely exceeds one year, as only Klotsche reported flare rates up until 48 months after discontinuation [20]. Long-term results of discontinuation are therefore still unclear. Despite these limitations the presented data in this review give an adequate representation of the current JIA population as data regarding relapse rates is comparable among the different studies.

Future studies are needed to show if *delayed remission* is indeed a risk factor for development of flares post bDMARD stoppage, as suggested by Su et al. [42]. A large multicentred cohort evaluating bDMARD discontinuation in JIA patients, using the same definitions for inactive disease, could provide more insight into this topic and might identify other possible flare related variables. We furthermore encourage future studies to display finding in a verifiable manner using uniform definitions and with a separate analysis of soJIA and non-systemic JIA subgroups.

In addition to patient characteristics and variables, biomarkers might also be helpful in predicting successful discontinuation [18, 23, 56]. Furthermore, ultrasound guided discontinuation is suggested as a mode to reduce flares after discontinuation, however studies outcomes are conflicting [57–60]. In summary, still more research needs to be conducted before a biomarker/ultrasound-based discontinuation strategy will be ready to be implemented.

In conclusion, this review showed that 60 to 78% of non-systemic JIA patients flare within one year after discontinuation, with a mean time to relapse of 2 to 8.4 months. Flares could not be reliably predicted by any predetermined variable at this point in time, mainly due to a lack of sufficient studies that primarily focussed on relapse rates and associated variables. This is the first study summarizing data on relapse rate and associated variables in non-systemic JIA patients after withdrawal of bDMARDs. Our current overview highlights the importance of future research and identifies several focus points for these studies. However this review shows that discontinuation of bDMARDs is feasible in JIA patients in general, and can be of assistance in daily practise in informing JIA patients and their parents on discontinuation of biologic therapy.

## Supplementary Information

**Additional file 1.** 

**Additional file 2.** 

**Additional file 3.**

## Data Availability

All data generated or analysed during this study are included in this published article and its supplementary information files.

## Abbreviations

bDMARDs: Biologic disease-modifying antirheumatic drugs
JIA: Juvenile idiopathic arthritis
soJIA: Systemic onset JIA
TTR: Time to relapse
RA: Rheumatoid arthritis
ETN: Etanercept
ANA: Antinuclear Antibodies
RF: Rheumatoid factor
MTX: Methotrexate
TNFi: Tumor Necrosis Factor Inhibitor
HR: Hazard Ratio
