# Supplementary material for: Discontinuation of biologic DMARDs in non-systemic JIA patients: a scoping review of relapse rates and associated factors
Source: Pediatr Rheumatol Online J. 2022 Dec 5;20:109. doi: 10.1186/s12969-022-00769-5 (PMC9721079; doi:10.1186/s12969-022-00769-5)
Supplement: Supplementary file 1 — Additional file 1. [file 12969_2022_769_MOESM1_ESM.zip › Supplement 1b. PRISMA-ScR-Checklist.docx]

**PRISMA-ScR Checklist**

1. **Title**

Review reported as scoping review in the title

1. **Abstract**

Abstract provided

**Intro**

1. Rationale is described in the introduction. The explicit reason the review questions lend themselves to a scoping review, is that this subject has not yet been comprehensively reviewed, and due to its broad and complex nature and the heterogeneousity it is not amenable to a more precise systematic review
2. Objectives are shown in het methods

**Methods**

1. This checklist has been indicated in the first paragraph of the methods
2. Specification of characteristics are shown in the “search strategy and eligibility of the studies” section of the methods. Rationale for excluding systemic JIA is given in the second paragraph if the discussion
3. Shown in the “search strategy and eligibility of the studies” section of the methods
4. Shown in the “search strategy and eligibility of the studies” section of the methods and supplement 2
5. Shown in the “search strategy and eligibility of the studies” section of the methods
6. Methods of charting data is shown in the “Data selection” section of the methods. Charting was done independently by the first author
7. Definition of flare is shown in the “Data selection” section of the methods
8. Not applicable
9. Definition of flare is shown in the “Data selection” section of the methods

**Results**

1. Shown in “search results” section of the results and in figure 1
2. Shown in “data selection”, results and in tables 1 and 2
3. Not applicable
4. Shown in results, tables 1 and 2 and figures 2, 3 and supplement 3
5. Shown in results, tables 1 and 2 and figures 2, 3 and supplement 3

**Discussion**

1. Shown in the discussion, primarily in the first and last paragraph
2. Shown in the fourth paragraph of the discussion
3. Shown in the remaining paragraphs of the discussion

**Funding**

1. Shown in the funding section
