# Supplementary material for: Discontinuation of biologic DMARDs in non-systemic JIA patients: a scoping review of relapse rates and associated factors
Source: Pediatr Rheumatol Online J. 2022 Dec 5;20:109. doi: 10.1186/s12969-022-00769-5 (PMC9721079; doi:10.1186/s12969-022-00769-5)
Supplement: Supplementary file 2 — Additional file 2. [file 12969_2022_769_MOESM2_ESM.docx]

***Supplement***

**Search strategy**

*Search terms used*

***JIA***:

1. "Arthritis, Juvenile"[Mesh], jia[tiab], jra[tiab]
2. Juvenile*[tiab] OR child*[tiab] Or pediatric*[tiab] OR paediatric*[tiab]

AND

rheumatoid[tiab] OR rheumatic[tiab] OR idiopathic[tiab] OR chronic[tiab]

AND

arthritis[tiab] OR arthritides[tiab] OR polyarthritis[tiab] OR oligoarthritis[tiab]

***bDMARDs:***

Biosimilar Pharmaceuticals[Mesh], biologics[tiab], biologic therapies[tiab], biologic therapy[tiab]

"Tumor Necrosis Factor Inhibitors"[Mesh], Anti-Tumor Necrosis Factor Therapy[tiab], anti-TNF therapy[tiab], TNF-alpha-blocking agents[tiab], tumor necrosis factor inhibitor[tiab], TNFi[tiab]

***Adalimumab:***

Adalimumab[Mesh], Adalimumab[tiab], humira[tiab], Amgevita[tiab], Hulio[tiab], Hyrimoz[tiab], Idacio[tiab], Imraldi[tiab]

***Etanercept:***

Etanercept[Mesh], Etanercept[tiab], enbrel[tiab], Benepali[tiab], Erelzi[tiab]

***Infliximab:***

Infliximab[Mesh], Infliximab[tiab], Remicade[tiab], monoclonal antibody cA2[tiab], Flixabi[tiab], Inflectra[tiab], Remsima[tiab], Zessly[tiab]

***Golimumab:***

Golimumab[Supplementary Concept], golimumab[tiab])) OR Simponi [tiab]

***Abatacept:***

Abatacept[Mesh] OR Abatacept[tiab] OR Orencia[tiab]

***Tocilizumab:***

Tocilizumab[Supplementary Concept], tocilizumab[tiab], Actemra[tiab], Atlizumab[tiab], RoActemra[tiab]

***Complete search***:

(("juvenile*"[Title/Abstract] OR "child*"[Title/Abstract] OR "pediatric*"[Title/Abstract] OR "paediatric*"[Title/Abstract]) AND ("rheumatoid"[Title/Abstract] OR "rheumatic"[Title/Abstract] OR "idiopathic"[Title/Abstract] OR "chronic"[Title/Abstract]) AND ("arthritis"[Title/Abstract] OR "arthritides"[Title/Abstract] OR "polyarthritis"[Title/Abstract] OR "oligoarthritis"[Title/Abstract]) AND ("Tumor Necrosis Factor Inhibitors"[MeSH Terms] OR "anti tumor necrosis factor therapy"[Title/Abstract] OR "anti tnf therapy"[Title/Abstract] OR "tnf alpha blocking agents"[Title/Abstract] OR "tumor necrosis factor inhibitor"[Title/Abstract] OR "TNFi"[Title/Abstract] OR ("Adalimumab"[MeSH Terms] OR "Adalimumab"[Title/Abstract] OR "humira"[Title/Abstract] OR "Amgevita"[Title/Abstract] OR "Hulio"[Title/Abstract] OR "Hyrimoz"[Title/Abstract] OR "Idacio"[Title/Abstract] OR "Imraldi"[Title/Abstract]) OR ("Etanercept"[MeSH Terms] OR "Etanercept"[Title/Abstract] OR "enbrel"[Title/Abstract] OR "Benepali"[Title/Abstract] OR "Erelzi"[Title/Abstract]) OR ("Infliximab"[MeSH Terms] OR "Infliximab"[Title/Abstract] OR "Remicade"[Title/Abstract] OR "monoclonal antibody cA2"[Title/Abstract] OR "Flixabi"[Title/Abstract] OR "Inflectra"[Title/Abstract] OR "Remsima"[Title/Abstract] OR "Zessly"[Title/Abstract]) OR ("golimumab"[Supplementary Concept] OR "golimumab"[Title/Abstract] OR "Simponi"[Title/Abstract]) OR ("Abatacept"[MeSH Terms] OR "Abatacept"[Title/Abstract] OR "Orencia"[Title/Abstract]) OR ("tocilizumab"[Supplementary Concept] OR "tocilizumab"[Title/Abstract] OR "Actemra"[Title/Abstract] OR "Atlizumab"[Title/Abstract] OR "RoActemra"[Title/Abstract]) OR ("Biosimilar Pharmaceuticals"[MeSH Terms] OR "biologics"[Title/Abstract] OR "biologic therapies"[Title/Abstract] OR "biologic therapy"[Title/Abstract]))) NOT (("juvenile*"[Title/Abstract] OR "child*"[Title/Abstract] OR "pediatric*"[Title/Abstract] OR "paediatric*"[Title/Abstract]) AND ("rheumatoid"[Title/Abstract] OR "rheumatic"[Title/Abstract] OR "idiopathic"[Title/Abstract] OR "chronic"[Title/Abstract]) AND ("arthritis"[Title/Abstract] OR "arthritides"[Title/Abstract] OR "polyarthritis"[Title/Abstract] OR "oligoarthritis"[Title/Abstract]) AND ("Tumor Necrosis Factor Inhibitors"[MeSH Terms] OR "anti tumor necrosis factor therapy"[Title/Abstract] OR "anti tnf therapy"[Title/Abstract] OR "tnf alpha blocking agents"[Title/Abstract] OR "tumor necrosis factor inhibitor"[Title/Abstract] OR "TNFi"[Title/Abstract] OR ("Adalimumab"[MeSH Terms] OR "Adalimumab"[Title/Abstract] OR "humira"[Title/Abstract] OR "Amgevita"[Title/Abstract] OR "Hulio"[Title/Abstract] OR "Hyrimoz"[Title/Abstract] OR "Idacio"[Title/Abstract] OR "Imraldi"[Title/Abstract]) OR ("Etanercept"[MeSH Terms] OR "Etanercept"[Title/Abstract] OR "enbrel"[Title/Abstract] OR "Benepali"[Title/Abstract] OR "Erelzi"[Title/Abstract]) OR ("Infliximab"[MeSH Terms] OR "Infliximab"[Title/Abstract] OR "Remicade"[Title/Abstract] OR "monoclonal antibody cA2"[Title/Abstract] OR "Flixabi"[Title/Abstract] OR "Inflectra"[Title/Abstract] OR "Remsima"[Title/Abstract] OR "Zessly"[Title/Abstract]) OR ("golimumab"[Supplementary Concept] OR "golimumab"[Title/Abstract] OR "Simponi"[Title/Abstract]) OR ("Abatacept"[MeSH Terms] OR "Abatacept"[Title/Abstract] OR "Orencia"[Title/Abstract]) OR ("tocilizumab"[Supplementary Concept] OR "tocilizumab"[Title/Abstract] OR "Actemra"[Title/Abstract] OR "Atlizumab"[Title/Abstract] OR "RoActemra"[Title/Abstract]) OR ("Biosimilar Pharmaceuticals"[MeSH Terms] OR "biologics"[Title/Abstract] OR "biologic therapies"[Title/Abstract] OR "biologic therapy"[Title/Abstract])) AND ("review"[Publication Type] OR "systematic review"[Filter]))

Filters applied: Dutch, English, French, German, Child: birth-18 years
