# Supplementary figures and images for: Discontinuation of biologic DMARDs in non-systemic JIA patients: a scoping review of relapse rates and associated factors
Source: Pediatr Rheumatol Online J. 2022 Dec 5;20:109. doi: 10.1186/s12969-022-00769-5 (PMC9721079; doi:10.1186/s12969-022-00769-5)

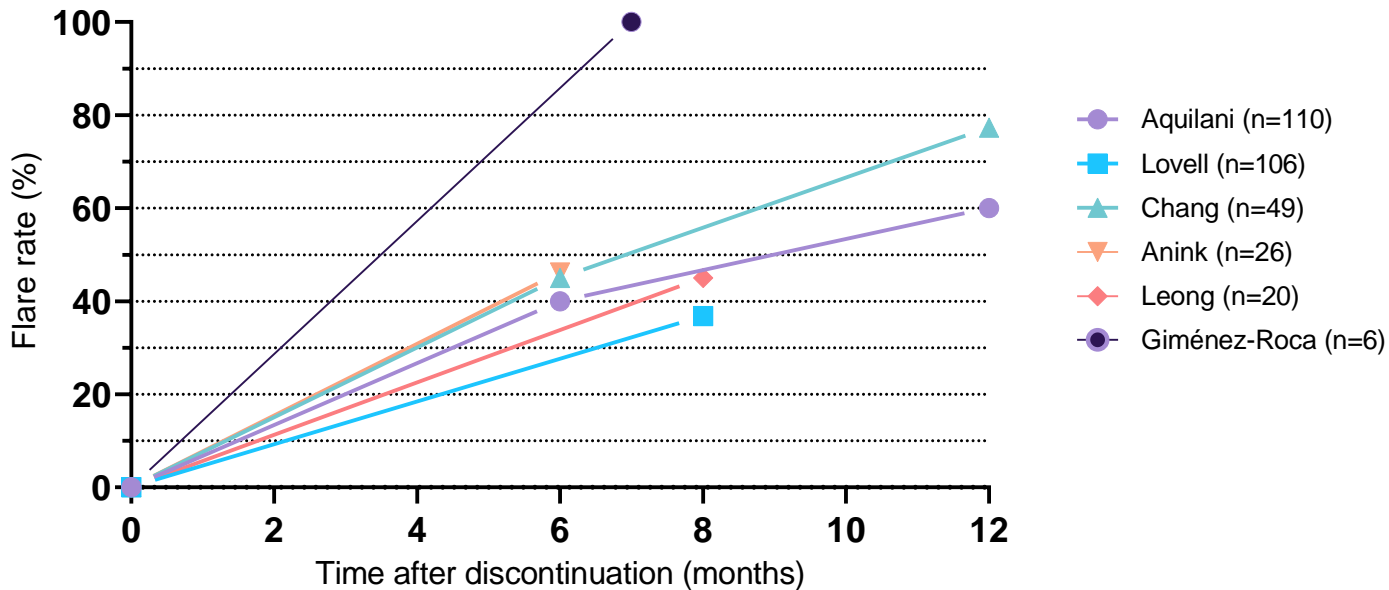

Supplement: Supplementary file 3 — Additional file 3. [file 12969_2022_769_MOESM3_ESM.pdf]
